# Genome-Wide Characterization of HERV-Derived RNA Regulatory Potential in the Human Transcriptome

**DOI:** 10.34133/csbj.0244

**Published:** 2026-11-05

**Authors:** Tomàs Montserrat-Ayuso, Aurora Pujol, Anna Esteve-Codina

**Affiliations:** ^1^ Centre Nacional d’Anàlisi Genòmica (CNAG), 08028 Barcelona, Spain.; ^2^ Universitat de Barcelona (UB), Barcelona, Spain.; ^3^ Neurometabolic Diseases Laboratory, Bellvitge Biomedical Research Institute (IDIBELL), Barcelona, Spain.; ^4^ Centre for Biomedical Research on Rare Diseases (CIBERER), Instituto de Salud Carlos III, Madrid, Spain.; ^5^ Catalan Institution of Research and Advanced Studies (ICREA), Barcelona, Spain.

## Abstract

Human endogenous retroviruses (HERVs) comprise nearly 8% of the human genome, and their long terminal repeats (LTRs) are well-established sources of transcriptional regulatory elements. However, their contribution to RNA-level regulatory features remains largely unexplored. Here, we present a genome-wide characterization of the RNA regulatory potential of 53,176 HERV internal regions and 560,594 LTRs. We found 16.2% more predicted RNA-binding protein motif occurrences in HERV internal regions than expected after controlling for dinucleotide composition. At the subfamily level, overall motif abundance was elevated in HERVH but remained close to expectation in HERVK (HML-2). Conserved retroviral protein domains incorporated into transcripts were mostly located in exceptionally long terminal exons, approximately 7-fold longer than those of other multi-exon transcripts. HERV internal sequence overlapped 4,074 annotated long noncoding RNA genes and accounted for more than 80% of the gene body of 144, most of them functionally unannotated. Finally, 6,583 unique antisense LTRs at transcript termini formed SPARCS-like candidate configurations that could support antisense RNA pairing; protein-coding genes carrying them in 3′ untranslated regions showed enrichment for immune signaling and recombination-related terms. These results provide a genome-wide framework of candidate HERV-derived RNA regulatory features and transcript architectures for experimental testing.

## Introduction

Transposable elements (TEs) account for roughly 45% of the human genome [1] and are increasingly recognized as important contributors to gene regulation [2]. Human endogenous retroviruses (HERVs), a major class of TEs derived from ancient germline infections by exogenous retroviruses, comprise approximately 8% of the genome and represent a particularly rich source of regulatory sequences, including long terminal repeats (LTRs) and internal regions with distinct functional properties [3]. HERV-derived LTRs have been extensively co-opted as promoters and enhancers that shape gene expression programs across development, cellular differentiation, and disease [4,5]. By contrast, the internal regions of HERVs remain comparatively understudied, despite retaining signatures of their retroviral origin, including retroviral protein-domain sequence preserved to varying degrees, as measured by coverage of domain profile hidden Markov models (HMMs) [6]. Their potential contribution to RNA-level regulatory processes has not been systematically explored.

Following their integration, HERV sequences have undergone extensive mutation, recombination, and deletion, resulting in a spectrum of genomic structures ranging from near-intact proviruses to fragmented internal regions and solo LTRs generated through homologous recombination [7]. Canonical proviral insertions are organized as LTR–internal–LTR structures, in which the internal region contains retroviral coding genes, including *gag*, *pol*, and *env* [3]. Although most HERV loci have lost protein-coding capacity, many internal regions retain recognizable coding remnants and partial structural organization [6]. This residual architecture suggests that, beyond their evolutionary origin, HERV internal regions may preserve sequence features with potential functional relevance.

Beyond their structural persistence, HERV sequences have been repeatedly co-opted at both the regulatory and coding level. At the regulatory level, LTRs function as promoters and enhancers that can rewire transcriptional programs, particularly in developmental and immune contexts [8,9], with recent genome-wide analyses further supporting the contribution of embryo-regulatory LTRs such as LTR7/HERVH and MLT2A/HERVL to broad developmental regulatory networks [10]. At the coding level, a small number of HERV-derived genes have been retained and repurposed, most notably the syncytins—retroviral envelope proteins essential for placental development [11,12]. At the same time, dysregulated HERV activity has been associated with a range of pathological conditions, including cancer [13–15], neurological disorders [16,17], and autoimmune diseases [18]. These dual roles highlight the importance of distinguishing background HERV transcription from loci that retain functional regulatory or coding potential.

At the RNA level, HERV-derived sequences have the potential to carry diverse regulatory features. These include binding sites for RNA-binding proteins (RBPs) [19,20], sequences that influence RNA stability, localization, and translation [21–23], and motifs capable of acting as competing endogenous RNAs through microRNA (miRNA) sequestration [24,25]. Subfamily-specific RBP binding to HERV-derived RNA has been demonstrated experimentally, for example, for RBM4 [19], and RBPs show characteristic binding patterns on repeat-derived RNA sequences [20]. In addition, specific genomic configurations involving HERV-derived sequences may give rise to double-stranded RNA (dsRNA) structures capable of activating innate immune sensing pathways [26]. Importantly, these features are expected to operate within the context of host transcripts, including long noncoding RNAs (lncRNAs) and protein-coding genes [27]. Previous work has also described HERVH-derived lncRNAs and spliced chimeric transcripts [28]. Despite these possibilities, the extent to which sequence features with RNA regulatory potential occur across HERV internal regions genome-wide—and whether they differ systematically between subfamilies and loci—remains unclear.

A systematic characterization of RNA-associated regulatory features within HERV internal regions is currently lacking. In particular, it remains unclear whether these regions are enriched for predicted RBP motif occurrences relative to sequence-composition-matched expectations, whether motif profiles vary across HERV subfamilies, and how they are integrated into host transcript architectures such as lncRNAs and protein-coding genes. Specific genomic configurations involving HERV sequences have been shown to generate dsRNA and activate innate immune signaling, as exemplified by SPARCS (stimulated 3 prime antisense retroviral coding sequences), where antisense LTRs located in 3′ untranslated regions (UTRs) of interferon-stimulated genes produce dsRNA through bidirectional transcription [26]. However, the extent to which comparable antisense configurations occur more broadly across the genome, and how they relate to LTR sequence features and transcript context, remains unclear. Moreover, an integrative, genome-wide framework linking these distinct regulatory layers has not been established.

Here, we present a genome-wide characterization of RNA regulatory potential across 53,176 HERV internal regions and 560,594 HERV LTRs in the human genome. We integrate several layers of annotation to characterize HERV-derived RNA regulatory potential. These include predicted RBP motifs, HERV overlap with protein-coding and noncoding genes, conserved retroviral protein domains, HERV contribution to lncRNA gene bodies, and antisense LTR configurations at transcript termini. Our analysis identifies subfamily-specific predicted RBP motif profiles that persist after composition control, shows that conserved domains mostly occur within exceptionally long terminal exons, characterizes a class of lncRNA genes whose gene bodies are dominated by HERV sequence, and catalogs widespread SPARCS-like candidate configurations of antisense LTRs at transcript termini. Together, these analyses provide a genome-wide map of candidate HERV-derived RNA regulatory features for experimental follow-up. To facilitate further exploration and reuse, all annotations generated in this study are provided as an openly accessible resource via Zenodo (https://doi.org/10.5281/zenodo.23361077).

## Results

### Global overview of predicted RNA regulatory features in HERV internal regions

To systematically characterize the regulatory potential of HERV internal regions at the RNA level, we integrated multiple layers of annotation across the human genome, including predicted RBP motifs, transcript overlap with coding and noncoding genes, and our previously generated annotations of conserved retroviral protein domains and LTR regulatory architecture [5,6].

This integrated framework enabled the joint analysis of sequence-derived features together with their genomic and annotated transcript context, allowing both subfamily-level comparisons and locus-specific interrogation.

At a global level, predicted RBP motifs were widespread across HERV internal regions. Overall, 88.5% of loci (47,076/53,176) contained at least one predicted motif at a FIMO threshold of *P* ≤ 10^−4^. To determine whether this prevalence exceeded that expected from sequence length and nucleotide composition, we generated 5 independently shuffled versions of each HERV internal region while preserving sequence length and exact dinucleotide frequencies, and repeated the motif scan using identical parameters. The proportion of shuffled loci containing at least one motif was similarly high, averaging 88.1% across replicates (range, 87.8% to 88.4%), indicating that the occurrence of at least one short RBP motif is largely expected in sequences of comparable length and composition.

By contrast, overall motif abundance was elevated in the observed HERV sequences. The original internal regions contained 716,075 predicted motif occurrences, compared with a mean of 616,109 across the shuffled controls. This corresponded to global motif densities of 12.77 and 10.98 occurrences per kilobase, respectively, representing a 16.2% excess over the dinucleotide-preserving expectation. To assess whether this global excess was also reflected at the locus level, we compared the motif density of each HERV internal region with the mean density of its 5 matched shuffled controls. Median locus-level motif density was higher in the observed sequences than in the matched shuffled controls (10.92 versus 9.77 occurrences per kilobase; paired Wilcoxon test, *P* = 3.78 × 10^−140^). These results indicate that, although predicted RBP motifs are intrinsically common in genomic sequences of comparable length and composition, HERV internal regions exhibit a modest but systematic excess of motif occurrences beyond that expected from dinucleotide composition alone.

We next examined whether this excess motif burden was distributed uniformly across HERV subfamilies or instead reflected subfamily-specific motif profiles.

### Composition-controlled analysis reveals subfamily-specific predicted RBP motif profiles

Using the same dinucleotide-preserving shuffled controls, we next asked whether composition-controlled motif profiles differed across HERV subfamilies (Table S1). For each subfamily and RBP, observed motif burden was compared with its matched shuffled expectation.

The composition-controlled motif profiles varied across HERV subfamilies. We focused on HERVH and HERVK (HML-2), which showed partially contrasting patterns of RBP motif enrichment and depletion (Fig. 1A). At the aggregate level, HERVH showed a higher motif burden than its composition-matched expectation (12.15 versus 9.99 occurrences/kb; observed/expected ratio = 1.216). By contrast, HERVK showed little difference from its shuffled expectation (10.56 versus 10.50 occurrences/kb; observed/expected ratio = 1.005). We next examined RBP-specific enrichment and depletion.

**Fig. 1.**
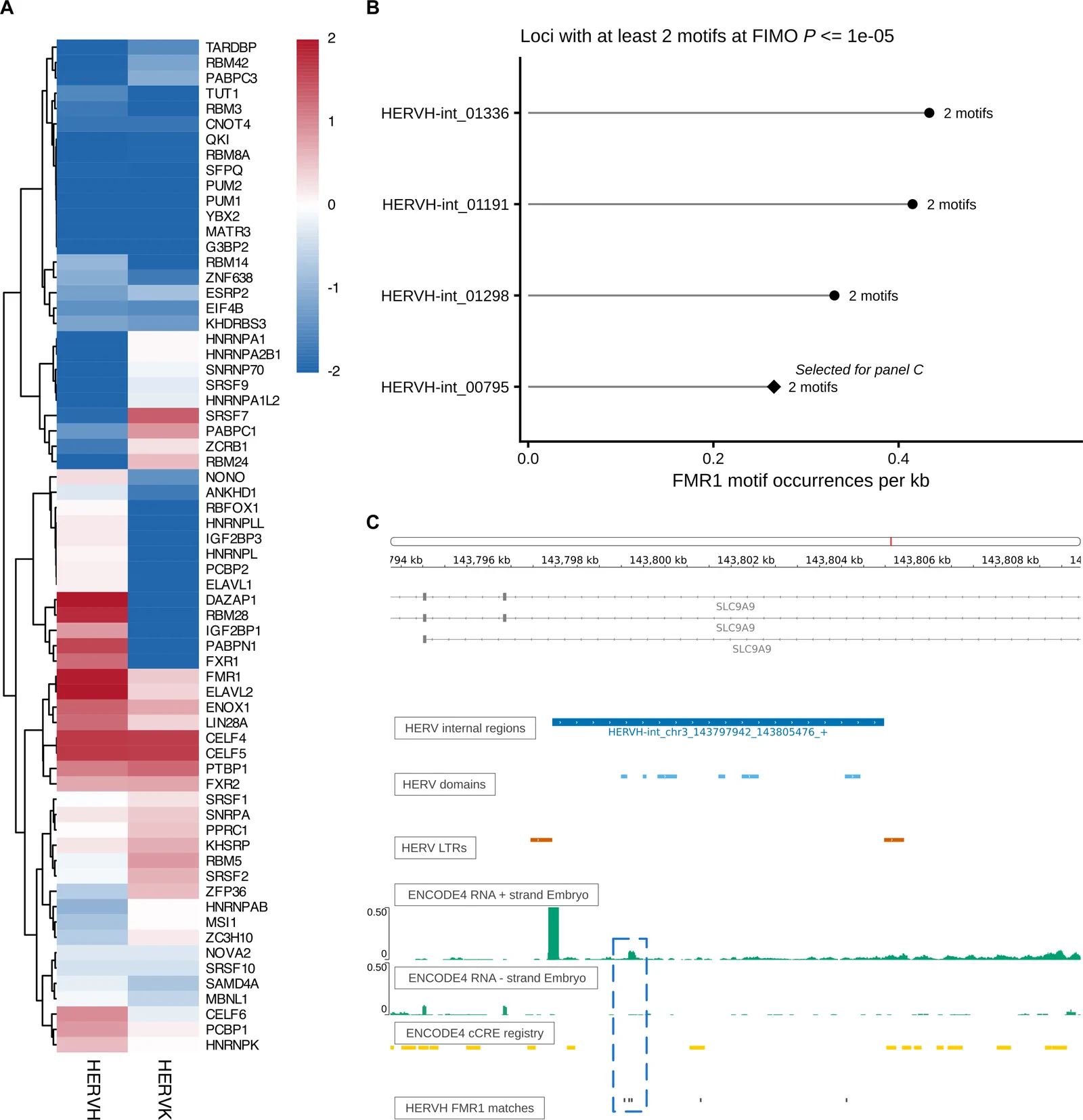
Subfamily- and locus-specific predicted RBP motif profiles across HERV internal regions. (A) Composition-controlled RBP motif profiles in HERVH and HERVK internal regions. Colors indicate log_2_ observed/expected motif abundance relative to 5 dinucleotide-preserving shuffled controls for RBPs significant in at least one subfamily (global adjusted *P* < 0.05); the color scale is capped at ±2. (B) FMR1 motif density among the 4 HERVH loci containing at least 2 predicted motifs at *P* ≤ 10^−5^. HERVH-int_00795 was selected for integrative inspection because it was the only candidate with detected embryonic RNA signal in the HERV orientation and additional epigenomic evidence. (C) Genomic context of HERVH-int_00795, located antisense within an intron of *SLC9A9*. Tracks show HERV structure, embryonic strand-specific RNA-seq signal, candidate cis-regulatory elements (cCREs), and predicted FMR1 motifs. The highlighted region contains FMR1 motifs. Visualization is generated using the freely accessible HERVarium web application (https://hervarium.cnag.eu/).

At the primary FIMO threshold of *P* ≤ 10^−4^, 20 of the 93 RBPs represented in the tested motif library were significantly enriched and 40 significantly depleted in HERVH, whereas 15 were enriched and 37 depleted in HERVK after global Benjamini–Hochberg (BH) correction (adjusted *P* < 0.05; Table S1). The heatmap in Fig. 1A shows composition-controlled log_2_ observed/expected values for the 66 RBPs that were significant in at least one of the 2 subfamilies. The resulting profiles were partially reciprocal: 6 RBPs—DAZAP1, FXR1, IGF2BP1, PABPN1, PCBP2, and RBM28—were enriched in HERVH and depleted in HERVK, whereas 7—PABPC1, RBM24, RBM5, SRSF1, SRSF2, SRSF7, and ZFP36—showed the opposite pattern. A further 8 RBPs, including PTBP1, KHSRP, FMR1, FXR2, and SNRPA, were significantly enriched in both subfamilies relative to their respective shuffled controls.

HERVH-enriched motifs included those recognized by LIN28A, IGF2BP1, ELAVL2, DAZAP1, HNRNPK, PCBP1, PCBP2, and FXR1. A dedicated Gene Ontology (GO) Biological Process (BP) overrepresentation analysis of the 20 significantly enriched HERVH RBPs, using all 93 tested motif-library RBPs as the background universe, did not identify any terms that remained significant after multiple-testing correction. The corresponding analysis of the 15 HERVK-enriched RBPs also yielded no significant terms.

To evaluate functional shifts across the complete motif profiles without restricting the analysis to individually significant RBPs, we additionally performed a pre-ranked GO BP analysis using all 93 tested RBPs. RBPs were ranked according to the difference between their composition-controlled log_2_ observed/expected values in HERVH and HERVK. This analysis identified significant enrichment of mRNA splicing, via spliceosome toward the HERVK-biased end of the ranking (normalized enrichment score = −2.04, adjusted *P* = 0.004; Table S2). The RBPs contributing most strongly to this enrichment included SRSF7, RBM42, HNRNPA1L2, SNRNP70, HNRNPA1, HNRNPA2B1, PABPC1, ZCRB1, RBM8A, and RBM5. No GO BP term was significantly enriched toward the HERVH-biased end after multiple-testing correction.

To assess sensitivity to motif-calling stringency, we repeated the analysis at *P* ≤ 10^−5^ (Table S3), compared with *P* ≤ 10^−4^ in the primary analysis. The aggregate motif excess remained evident (13,702 observed versus 12,231 expected; observed/expected = 1.120), with a larger excess in HERVH (2,470 versus 1,138; observed/expected = 2.171) and a smaller excess in HERVK (148 versus 114; observed/expected = 1.296). However, the stricter threshold retained only ~2% of the original motif calls, reducing the number of predicted motifs per RBP and altering enrichment results. GO overrepresentation analysis of HERVH-enriched RBPs identified a single significant term (regulation of multicellular organismal process; adjusted *P* = 0.025). In contrast, no GO BP term reached significance in the pre-ranked analysis, including the HERVK-biased mRNA splicing, via spliceosome signal observed at the primary threshold.

Together, these analyses revealed partially reciprocal HERVH and HERVK motif profiles, while GO enrichment results were sensitive to motif-calling stringency.

### FMR1 motif enrichment guides prioritization of a transcriptionally supported HERVH locus within *SLC9A9*

To illustrate how composition-controlled subfamily-level motif enrichment can guide locus prioritization, we focused on FMR1. FMR1 motifs were strongly enriched in HERVH relative to dinucleotide-preserving shuffled controls at both the primary and stricter FIMO thresholds (log_2_ observed/expected = 1.95 and 3.36; global BH-adjusted *P* = 2.34 × 10^−183^ and 1.50 × 10^−90^, respectively).

We next screened individual HERVH loci for predicted FMR1 motifs retained at the stricter threshold. Four loci each contained 2 such occurrences (Fig. 1B). We compared these 4 loci using the same embryo-associated RNA and epigenomic annotations available through HERVarium. HERVH-int_00795 (chr3:143,797,942–143,805,476) was the only candidate with detected embryonic RNA signal in the HERV orientation, together with H3K27ac evidence; the other 3 candidates showed no detected signal in the corresponding embryo-specific tracks. We therefore selected HERVH-int_00795 for integrative inspection based on the convergence of strict-threshold motif predictions and independent transcriptional evidence.

HERVH-int_00795 contains both 5′ and 3′ LTRs, retains partial *gag*, *pol*, and *env* domain signatures, and lies antisense within an intron of the protein-coding gene *SLC9A9* (Fig. 1C). Strand-specific embryonic RNA-sequencing (RNA-seq) signal was detected in the HERV orientation rather than the *SLC9A9* orientation. Apart from a prominent transcriptional signal near the 5′ end of the insertion, a second clear increase in same-strand RNA-seq coverage was observed farther within the locus, with signal intensity comparable to that detected over nearby *SLC9A9* exons. This region overlapped one strict-threshold FMR1 motif and one additional motif retained at *P* ≤ 10^−4^, while the second strict-threshold motif was located immediately upstream. Multiple candidate cis-regulatory elements were also distributed across the locus. Together, the composition-controlled HERVH enrichment, strict-threshold motif predictions, same-strand embryonic RNA signal, and regulatory annotations prioritize HERVH-int_00795 as a candidate transcriptionally active HERVH locus carrying predicted FMR1 motifs.

### Conserved HERV-derived domains are associated with terminal-exon architecture

To place HERV internal regions within their transcriptional context, we integrated HERV annotations with GENCODE gene models across the human genome. Overall, 11,397 HERV internal regions overlapped at least one annotated lncRNA on the same strand, corresponding to 4,074 unique lncRNA genes, whereas 3,514 regions overlapped protein-coding genes on the same strand, involving 1,521 unique genes. Intronic overlaps predominated in both gene classes, accounting for 20.7% (11,001) of all HERV internal regions in lncRNAs and 6.3% (3,352) in protein-coding genes. Exonic incorporation was less frequent, involving 2,435 HERV loci in lncRNAs and 434 loci in protein-coding genes, corresponding to approximately 4.6% and 0.8% of all HERV internal regions, respectively (Fig. 2A). These categories are not mutually exclusive because the same HERV locus may overlap intronic and exonic regions in different transcript isoforms.

**Fig. 2.**
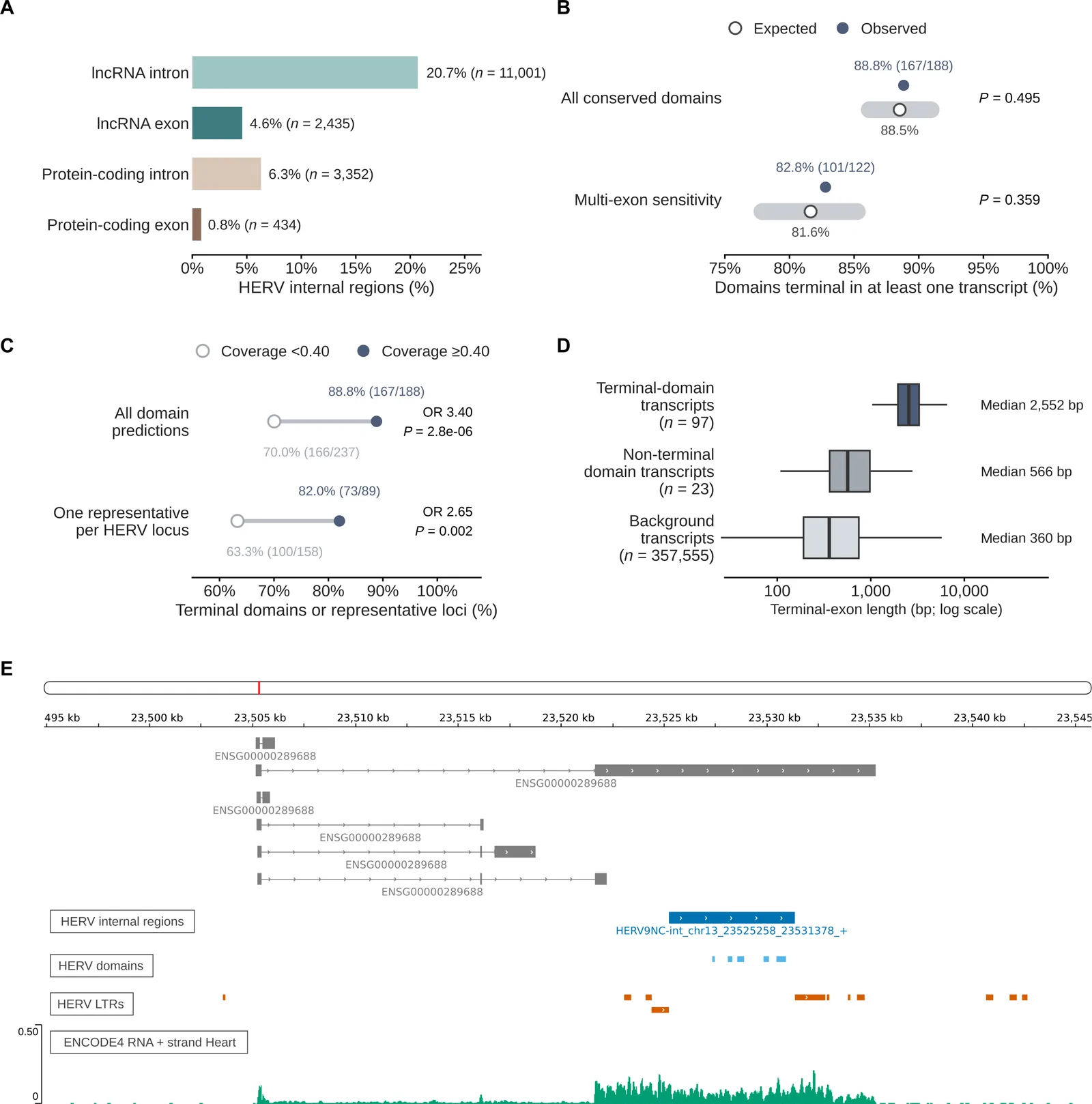
Terminal localization of HERV-derived domains is compatible with transcript architecture and associated with higher HMM profile coverage. (A) Same-strand overlap of HERV internal regions with lncRNA and protein-coding introns and exons, shown as the percentage and number of all 53,176 HERV internal regions; categories are not mutually exclusive. (B) Observed terminal localization of the 188 conserved domains, and of the 122 domains represented in multi-exon transcripts, compared with a transcript-aware positional null expectation; gray intervals show the exact 95% null interval. Observed values lie within the null ranges (*P* = 0.495 and *P* = 0.359, respectively), with no significant positional enrichment. (C) Terminal localization of conserved (HMM profile coverage ≥0.40) and lower-coverage (<0.40) domain predictions, considering all exon-overlapping domains and, separately, one representative domain per HERV locus. Odds ratios (ORs) and Fisher’s exact test *P* values are shown. (D) Terminal-exon length distributions of multi-exon transcripts grouped according to HERV-derived domains with HMM profile coverage ≥0.40: terminal-exon domain (*n* = 97), nonterminal domain only (*n* = 23), or background transcripts from genes without exon-overlapping conserved HERV-derived domains (*n* = 357,555). Medians are indicated; terminal-exon length is shown on a logarithmic scale. (E) HERVarium browser view of a terminal-exon HERV9NC insertion in lncRNA ENSG00000289688 containing multiple conserved domains and overlapping heart RNA-seq signal on the + strand (gene orientation).

We next focused on HERV-derived protein-domain predictions with at least 40% coverage of the corresponding HMM profile, hereafter termed conserved domains. Domains were considered incorporated into a transcript when at least 80% of their genomic length was covered by exonic sequence within a transcript. This reconstruction identified 259 domain–transcript associations involving 188 unique conserved genomic domains, 144 transcripts, 87 genes, and 89 HERV loci.

A single genomic domain can occur in more than one transcript isoform. Among the 188 conserved domains, 161 were terminal in all associated isoforms, 6 changed between terminal and nonterminal positions depending on the isoform, and 21 were nonterminal in every associated isoform. Thus, 167 of 188 domains were terminal in at least one transcript, corresponding to 88.8%. To exclude the special case of single-exon transcripts, in which the only exon is necessarily also terminal, we repeated the analysis among the 122 domains represented in at least one multi-exon transcript. In this subset, 101 domains, or 82.8%, were terminal in at least one multi-exon isoform.

We then tested whether this high terminal proportion exceeded the expectation generated by domain length and host-transcript architecture. For each domain, we evaluated every possible placement of a same-length interval within its relevant transcript context and retained only positions satisfying the same ≥80% exonic-overlap criterion used for the observed data. These valid positions were used to calculate a domain-specific probability of terminal localization. Of the 188 conserved domains, 167 were terminal, closely matching the 166.43 expected under the null model (95% null interval, 162 to 171; *P* = 0.495; Fig. 2B). Similar results were obtained for multi-exon transcripts (101 observed versus 99.59 expected; 95% null interval, 95 to 104; *P* = 0.359). Thus, the high proportion of terminal domains did not exceed architectural expectations, a conclusion that remained consistent across alternative localization criteria and transcript-isoform definitions.

Although the absolute terminal proportion was compatible with transcript architecture, higher HMM profile coverage remained associated with terminal localization. When the reconstruction was extended to all 425 exon-overlapping domain predictions, 88.8% of predictions with HMM profile coverage ≥0.40 were terminal, compared with 70.0% of lower-coverage predictions. This corresponded to an odds ratio of 3.40 [95% confidence interval (CI) 2.00 to 5.79; Fisher’s exact *P* = 2.8 × 10^−6^]. Because several domains can originate from the same HERV insertion and share the same transcript context, we repeated the comparison after selecting only the highest-coverage domain from each HERV locus. The association remained significant at this locus-controlled level: 82.0% of loci with a conserved representative domain were terminal, compared with 63.3% of loci represented by a lower-coverage domain (odds ratio 2.65, 95% CI 1.41 to 4.97; *P* = 0.0023; Fig. 2C). Thus, the association was not explained solely by repeatedly counting several domains from the same HERV insertion.

Although positional enrichment was not supported, the architecture of the host transcripts differed markedly. Among multi-exon transcripts, the 97 isoforms containing a conserved domain in their terminal exon had a median terminal-exon length of 2,552 base pairs (bp) (interquartile range 1,951 to 3,287 bp), approximately 7-fold longer than the median of 360 bp (191 to 745 bp) among the 357,555 multi-exon transcripts from genes without conserved HERV-derived domains (2-sided Mann–Whitney test; Cliff’s delta 0.858; BH-adjusted *P* = 6.31 × 10^−47^; Fig. 2D). Transcripts carrying conserved domains only in nonterminal exons (*n* = 23) had an intermediate median terminal-exon length of 566 bp. The median terminal exon accounted for 80.8% of the total exonic length of terminal-domain transcripts, compared with 42.7% in the background group. The association was strongest at the transcript-isoform level but remained significant when each gene was counted once. Genes containing terminal conserved domains had a median terminal-exon length across their multi-exon isoforms of 641 bp, compared with 363 bp in background genes (Cliff’s δ = 0.424; BH-adjusted *P* = 3.41 × 10^−7^), and their longest annotated terminal exon had a median length of 3,448 bp compared with 682 bp in background genes (Cliff’s δ = 0.757; BH-adjusted *P* = 1.55 × 10^−21^). These results indicate that terminal conserved domains are strongly associated with transcript isoforms characterized by unusually long terminal exons, with the association also evident at the gene level.

Representative protein-coding and lncRNA loci illustrate the diversity of these terminal-exon configurations. Protein-coding examples include established domesticated retroviral genes such as *ERVW-1* and *ERVFRD-1*, as well as *ERV3-1*, whose Env domain is embedded within the terminal exon of ZNF117. Among lncRNAs, ENSG00000289688 contains a HERV9NC insertion with protease, ribonuclease (RNase) H, reverse-transcriptase, and Env domain predictions, with HMM profile coverage values reaching 0.96. At this locus, strand-specific RNA-seq data from heart tissue also showed signal across the HERV-containing terminal exon in the annotated gene orientation, providing supporting evidence of transcription (Fig. 2E and Fig. S1A). Another lncRNA, ENSG00000285958, contains a full-length protease (AP) domain prediction (HMM profile coverage 1.0) and an RNase H domain prediction (coverage 0.6) within its terminal exon (Fig. S1B).

Together, these results show that conserved HERV-derived domains frequently occur in terminal exons without significant positional enrichment. Higher-coverage domains are more often terminal, and terminal-domain-containing transcripts have unusually long terminal exons.

### Quantitative overlap analysis identifies a class of HERV-dominated lncRNAs

#### HERV internal regions frequently contribute to lncRNA transcript architecture

To assess the contribution of HERV internal regions to annotated lncRNAs, we intersected the catalog of internal HERV loci with the genomic coordinates of human lncRNA genes. In total, 4,074 lncRNA genes were found to overlap at least one HERV internal region, indicating that retroviral sequences are common components of lncRNA loci.

However, the degree of retroviral contribution varied widely across genes. To quantify this variation, we calculated the fraction of each lncRNA gene body overlapping HERV internal sequence and classified genes according to the extent of HERV contribution.

The vast majority of HERV-associated lncRNAs contained only small retroviral fragments, with 3,043 lncRNA genes harboring HERV sequence across at most 10% of their gene body (Fig. 3A). In contrast, a smaller subset of loci showed substantial retroviral composition. Specifically, 750 lncRNA genes had more than 10% and up to 50% of their gene body covered by HERV sequence, while 137 genes showed 50% to 80% coverage. Notably, 144 lncRNA genes had more than 80% of their gene body covered by HERV internal sequence (Fig. 3B and Table S4). Because gene-body coverage includes introns, we also examined the merged exonic sequence of these genes: 39 of the 144 had more than 80% of their exonic sequence derived from HERV internal regions, whereas 70 had at most 50% (Table S4). The genomic span of these loci therefore largely coincides with retroviral insertions rather than representing host genes that incidentally contain small transposable-element fragments (Fig. 3C), although the retroviral contribution to their exonic sequence varies. Whether, and in which cell types, they are transcribed was not assessed systematically here.

**Fig. 3.**
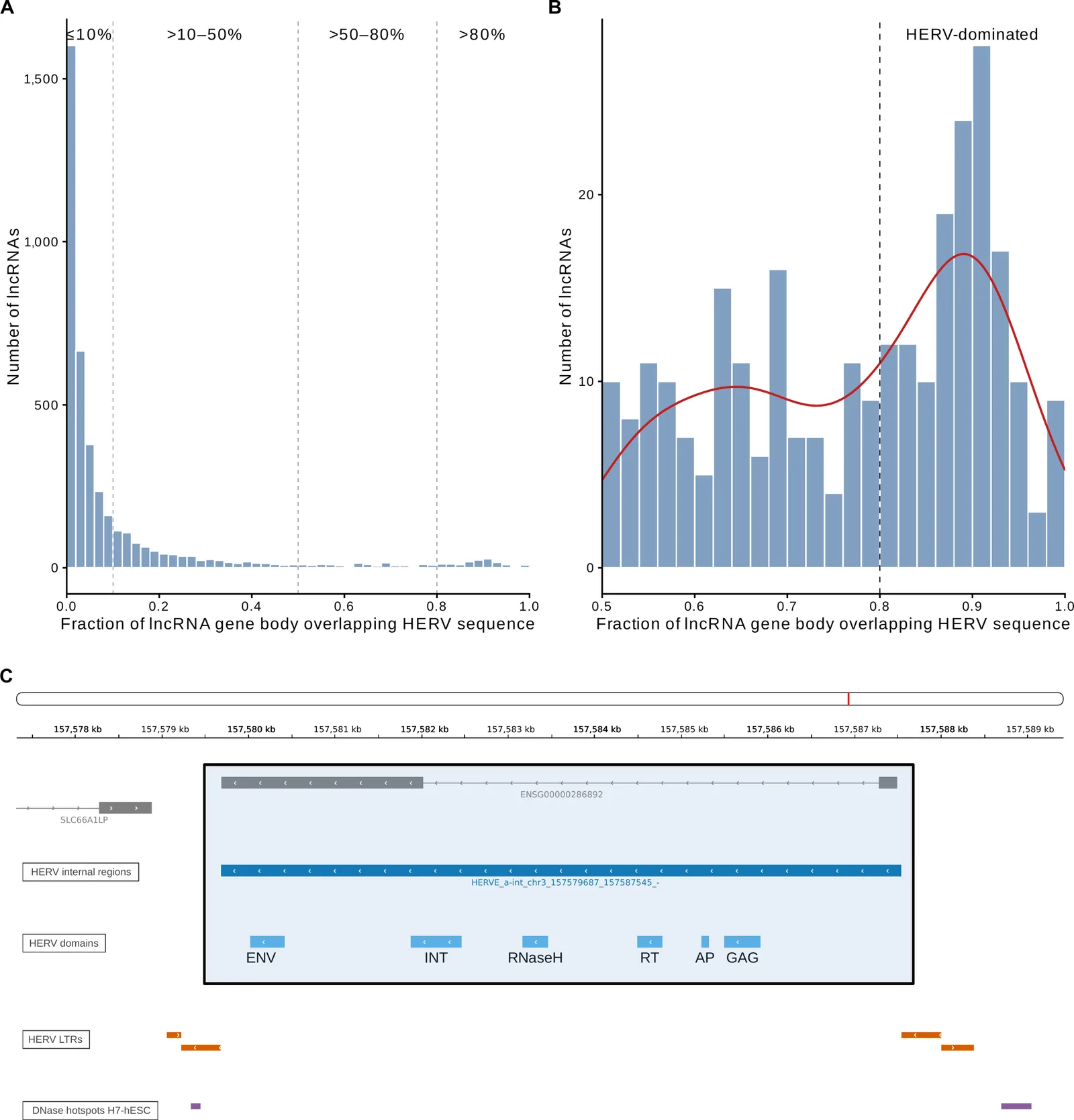
Quantitative contribution of HERV internal regions to lncRNA architecture reveals a subclass of HERV-dominated lncRNA genes. (A) Distribution of the fraction of the gene body overlapping HERV internal sequence for the 4,074 HERV-overlapping lncRNA genes. Most lncRNA genes contain only limited HERV-derived sequence and are concentrated at low coverage values. Dashed lines indicate the thresholds used to define the gene-body HERV contribution categories (≤10%; >10% to 50%; >50% to 80%; >80%). (B) Zoomed view of the 281 lncRNA genes with more than 50% gene-body HERV coverage. The dashed line marks the >80% gene-body threshold that defines the 144 HERV-dominated lncRNA genes. The density curve shows the distribution of gene-body coverage within this subset. (C) Illustrative genome-browser view of one HERV-dominated lncRNA locus (ENSG00000286892), whose entire gene body lies within a HERV internal region (HERVE_a); in this locus, the exonic sequence is also entirely HERV-derived. The locus contains multiple recognizable retroviral protein domains (Env, integrase, RNase H, reverse transcriptase, protease, and Gag) and is flanked by LTRs, illustrating how an endogenous retroviral insertion can form the primary structural unit of an annotated lncRNA gene. ENCODE deoxyribonuclease (DNase) I hotspots in H7 human embryonic stem cells flank the locus. Visualization is generated using the HERVarium web application (https://hervarium.cnag.eu/), with highlighting added for clarity.

We performed an analogous analysis in protein-coding genes. Although 1,521 protein-coding genes overlapped at least one HERV internal region, extensive HERV contribution was rare. The cases with the highest gene-body HERV coverage corresponded primarily to known co-opted retroviral genes, including *ERVW-1* (syncytin-1), *ERVH48-1* (suppressyn), and *ERVFRD-1* (syncytin-2), as well as a small number of additional loci with annotations related to retroviral envelope proteins (e.g., ENSG00000293569 and ENSG00000293570) (Table S5). In contrast to lncRNAs, we did not observe a substantial class of protein-coding genes dominated by HERV sequence, a difference that may reflect constraint on protein-coding sequence, although selection was not assessed here.

#### Functional annotation of HERV-associated lncRNAs is sparse

To evaluate whether HERV-associated lncRNA genes have existing functional annotation, we integrated curated annotations from the LncRNAWiki [29] database together with literature-derived functional descriptions obtained through automated mining of PubMed titles and abstracts.

Despite the large number of HERV-associated lncRNAs identified, functional annotation was limited. Only 593 of the 4,074 lncRNAs showed any functional description or literature association, while 3,481 lncRNA genes lacked any curated annotation, GO terms [30,31], or literature evidence, highlighting the largely unexplored nature of this gene class (Fig. 4A and Table S6).

**Fig. 4.**
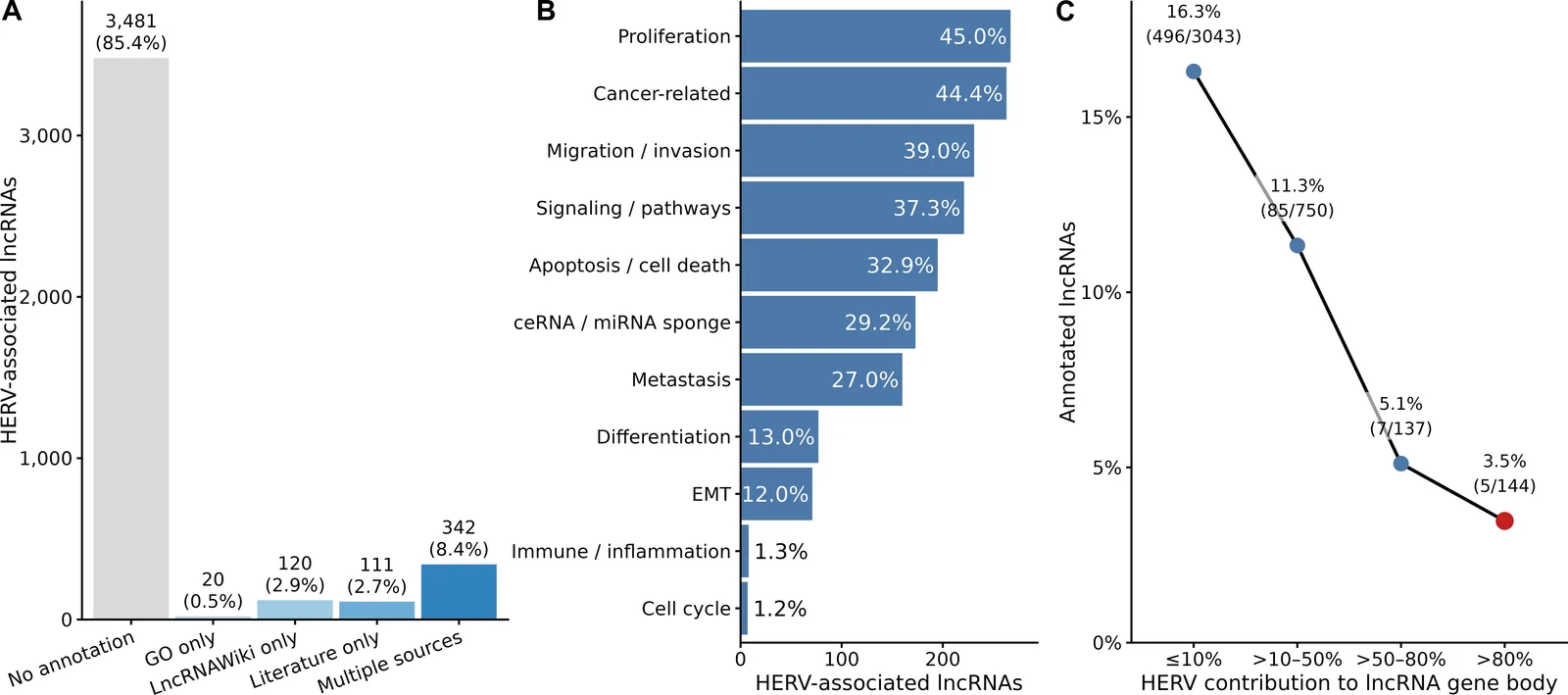
Functional annotation landscape of HERV-associated lncRNAs. (A) Distribution of functional annotation sources across HERV-associated lncRNAs. The majority of HERV-associated lncRNA genes lack any functional annotation (3,481 of 4,074; 85.4%), while a minority are supported by GO, LncRNAWiki, or literature-derived evidence. (B) Functional categories associated with annotated HERV-associated lncRNAs. Categories were derived by grouping curated LncRNAWiki and literature-mined annotations into broad, nonmutually exclusive biological processes. The most frequent associations include proliferation, cancer-related processes, migration/invasion, signaling, apoptosis, and ceRNA/miRNA sponge activity. (C) Relationship between HERV contribution and functional annotation. The proportion of annotated lncRNAs decreases with increasing gene-body HERV coverage, reaching 3.5% among HERV-dominated lncRNA genes.

Among the lncRNAs with functional annotation, reported biological associations were dominated by categories related to proliferation, cancer, migration/invasion, signaling, apoptosis, and metastasis. At the mechanistic level, one of the most recurrent annotated processes was competing endogenous RNA (ceRNA) activity, in which lncRNAs are proposed to act as miRNA sponges. In total, 173 HERV-associated lncRNAs were linked to ceRNA-related annotations (Fig. 4B).

Strikingly, functional information was almost entirely absent for the lncRNA genes with the highest gene-body HERV coverage. Of the 144 lncRNA genes whose gene body is more than 80% HERV internal sequence, only 5 genes (3.5%) showed any functional annotation or literature evidence (Fig. 4C). Among these, only 2 genes (*CERNA2* and *LINC02617*) had curated annotation in the LncRNAWiki database.

Together, these results indicate that while HERV sequences are widespread components of lncRNA loci, the biological roles of most of these lncRNA genes remain largely unexplored.

### Antisense LTR insertions at transcript termini form widespread SPARCS-like candidate configurations

Antisense HERV insertions at transcript termini could give rise to complementary RNA molecules capable of forming dsRNA through bidirectional transcription, which could trigger innate immune sensing pathways. Experimentally characterized examples, termed SPARCS (stimulated 3 prime antisense retroviral coding sequences), have been described in cancer cells as interferon-inducible HERVs located in the 3′ UTRs of specific genes, where bidirectional transcription generates dsRNA and triggers innate immune signaling [26].

To investigate whether similar genomic configurations are more broadly present in the human transcriptome, we systematically identified antisense LTR insertions located within the 3′ regions of annotated transcripts. Because these elements were identified based on their genomic configuration rather than experimentally demonstrated dsRNA formation, we refer to them as SPARCS-like candidates (Fig. 5A).

**Fig. 5.**
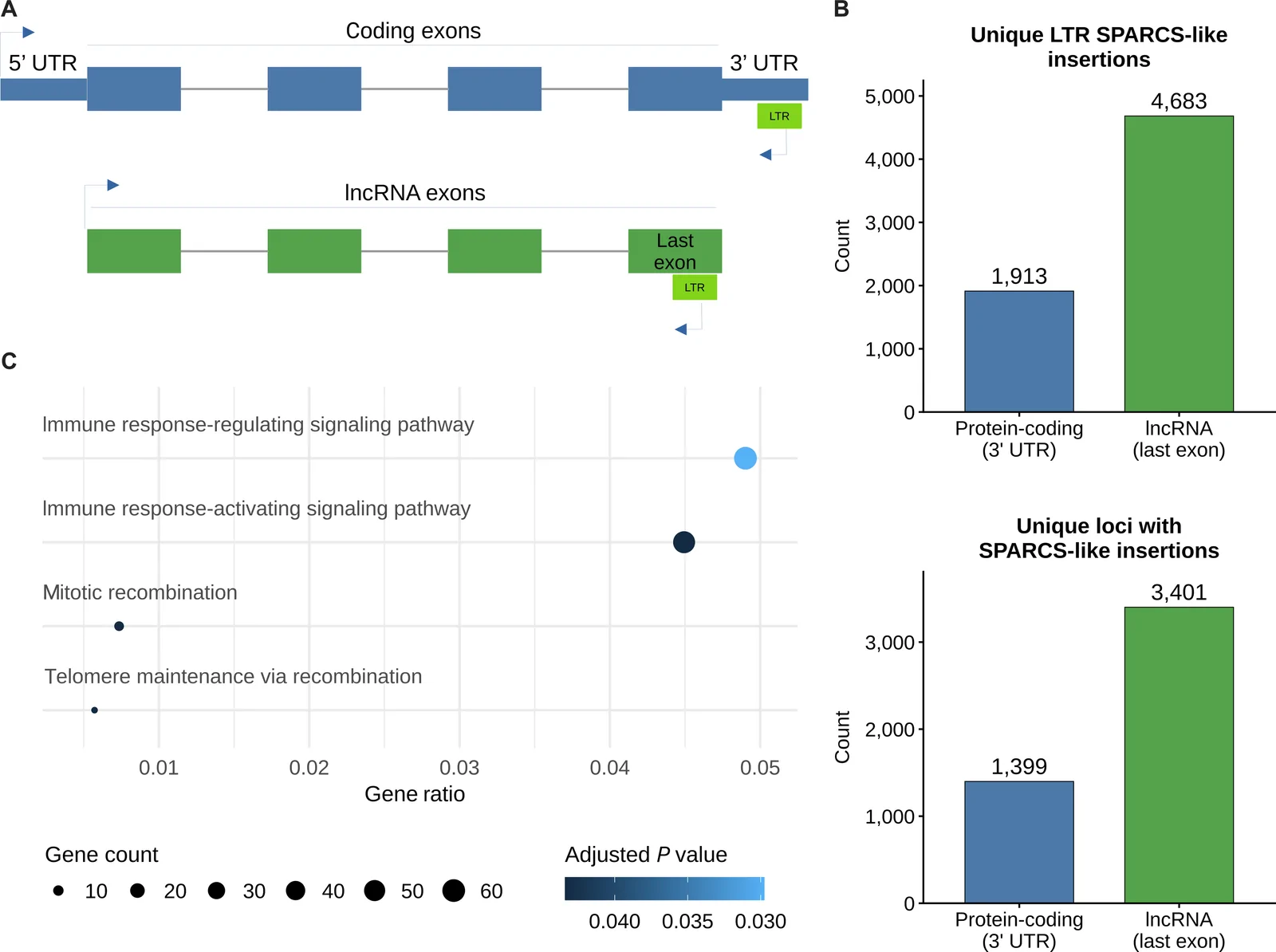
Antisense LTR insertions at transcript termini form widespread SPARCS-like candidate configurations. (A) Schematic representation of SPARCS-like candidate configurations. In protein-coding genes, antisense LTR insertions are located within 3′ UTRs, whereas in lncRNAs they are frequently embedded within the last exon. In both cases, the antisense orientation relative to the host transcript would allow complementary RNA pairing if both strands were transcribed. (B) Genome-wide quantification of SPARCS-like candidates. Top: Number of unique antisense LTR insertions located within transcript termini. Bottom: Number of affected genes. Antisense LTR insertions are more frequent in lncRNA last exons than in protein-coding 3′ UTRs, affecting a larger number of loci. (C) GO BP overrepresentation analysis of protein-coding genes harboring SPARCS-like insertions in their 3′ UTRs. Dot size represents gene count, and color indicates adjusted *P* value. The 4 significant terms comprise 2 immune signaling pathways and 2 recombination-related terms.

Across the human genome, we identified 1,913 antisense LTR insertions located within the 3′ UTRs of protein-coding genes, affecting 1,399 genes. In addition, we detected 4,683 antisense LTR insertions located within the last exons of lncRNAs, corresponding to 3,401 distinct lncRNA genes (Fig. 5B). In total, these analyses identify 6,583 unique antisense LTR insertions located within transcript termini (13 LTRs occur in both categories), indicating that SPARCS-like candidate configurations are widespread in the annotated human transcriptome.

GO BP overrepresentation analysis of the protein-coding genes harboring SPARCS-like candidates in their 3′ UTRs identified 4 significant terms: immune response-regulating signaling pathway, immune response-activating signaling pathway, mitotic recombination, and telomere maintenance via recombination (Fig. 5C and Table S7).

Overrepresentation analysis of the same genes against the immunologic signature (C7) collection of the Molecular Signatures Database (MSigDB) [32] identified 3 significantly enriched signatures: GSE42021_TREG_PLN_VS_CD24INT_TREG_THYMUS_DN, GSE23925_LIGHT_ZONE_VS_DARK_ZONE_BCELL_DN, and GSE42021_CD24HI_VS_CD24LOW_TCONV_THYMUS_DN (Table S7). Each comprises genes expressed at lower levels in the first than in the second of 2 immune-cell populations: regulatory T cells from peripheral lymph nodes compared with CD24-intermediate thymic regulatory T cells, germinal-center light-zone compared with dark-zone B cells, and CD24-high compared with CD24-low thymic conventional T cells, respectively. Among the genes from our candidate set contributing to both enriched thymic signatures were the interferon-stimulated genes OAS2, OASL, IFI44L, and TRIM22. These results place a subset of SPARCS-like candidate-containing genes within transcriptional programs that differ across immune-cell states.

Several protein-coding genes carrying SPARCS-like candidate LTRs in their 3′ UTRs also encode established components of innate immune sensing and interferon signaling pathways. Representative examples include the interferon-stimulated genes *OAS1*, *OAS3*, *DDX60*, *EIF2AK2* (*PKR*), and *GBP5*; pattern-recognition and antiviral signaling genes such as *TLR3* and *MAVS*; and additional immune regulators including *NLRP1*, *BIRC3*, *PTPRC* (*CD45*), and *LCP2* (*SLP-76*).

To determine whether SPARCS-like LTRs exhibit regulatory signatures associated with interferon responses, we examined the enrichment of interferon-responsive transcription factor binding motifs, specifically signal transducer and activator of transcription 1 (STAT1), STAT1::STAT2, and members of the IRF family [33–35]. SPARCS-like LTRs located in protein-coding 3′ UTRs showed no significant enrichment or depletion of these motifs relative to all other annotated LTRs, nor did they show enrichment of clustered STAT1/IRF motif configurations (all adjusted *P* = 1; Table S7).

Transcription factor motif analysis revealed further differences. In particular, motifs associated with developmental transcription factors—especially members of the HOX family and other patterning regulators—were significantly depleted in SPARCS-like LTRs located within lncRNA relative to all other scanned LTRs. An exploratory GO analysis of these depleted transcription factors highlighted biological processes related to regionalization and pattern specification (adjusted *P* ≤ 0.10; Table S8 and Fig. S2).

We next examined whether SPARCS-like LTRs display features indicative of increased sequence preservation. Using previously reconstructed canonical U3–R–U5 annotations [5], we did not observe increased structural integrity relative to other genomic LTRs, with similar proportions of loci exhibiting plausibly reconstructed structures (Table S7). Thus, we found no evidence that SPARCS-like candidate configurations are preferentially associated with structurally intact LTRs.

Together, these analyses identify widespread SPARCS-like candidate configurations at transcript termini. Protein-coding genes carrying these LTRs were enriched for immune-related annotations, although their LTRs showed no enrichment of interferon-responsive motifs. In lncRNAs, candidate LTRs showed depletion of developmental transcription factor motifs, while no evidence of increased structural integrity was found for SPARCS-like LTRs overall.

## Discussion

HERVs have traditionally been studied as sources of DNA-level regulatory elements, with particular emphasis on LTRs acting as promoters and enhancers that shape host transcriptional programs. Here, we extend this perspective to RNA-level regulatory potential by characterizing HERV-derived sequence features and their associations with host transcript architectures at genome-wide scale.

The composition-controlled analysis revealed a modest but systematic excess of predicted RBP motif occurrences in HERV internal regions, despite their high prevalence in both native and shuffled sequences. This excess varied across subfamilies: HERVH showed an elevated overall motif burden relative to its shuffled controls, whereas HERVK (HML-2) remained close to expectation. At the RBP level, the 2 subfamilies displayed partially contrasting enrichment and depletion patterns, potentially reflecting differences in sequence organization and evolutionary history.

These sequence-level patterns were not accompanied by robust functional enrichment of the corresponding RBP sets. Neither the HERVH- nor HERVK-enriched RBPs yielded significant GO BP terms, and the HERVK-biased spliceosome signal in the pre-ranked analysis was sensitive to motif-calling stringency. Thus, the motif profiles do not establish distinct RNA-regulatory programs. In light of previously documented interactions between HERV- and repeat-derived RNAs and RBPs [19,20], these motif profiles may help prioritize individual loci for experimental investigation when combined with independent transcriptional evidence, as illustrated by HERVH-int_00795.

The high proportion of HERV-derived domains found in terminal exons does not exceed that expected from domain length and host-transcript architecture. However, this does not resolve why terminal exons containing conserved HERV-derived domains are unusually long. Long terminal exons may be more permissive to retroviral sequence, HERV incorporation may itself contribute to exon length, or both may reflect other properties of these loci. More broadly, this association is consistent with the preferential accumulation of TE-derived sequence in relatively permissive transcript contexts [28,36–38]. Rather than supporting selection for terminal localization itself, our results reveal an association between terminal-exon architecture and the persistence of recognizable HERV-derived sequence within annotated transcripts, without establishing the direction of this relationship.

Nevertheless, the association between HERV-derived domains and terminal-exon architecture may have biological implications. Terminal exons, and particularly 3′ UTRs, are major sites of post-transcriptional regulation [39,40], so HERV-derived sequence embedded in these regions could influence RNA stability, localization, or translation if it is transcribed and functionally engaged. The observed relationship between higher HMM profile coverage and terminal localization may therefore help prioritize loci in which more complete retroviral domain signatures occur within potentially regulatory transcript contexts.

HERV-dominated lncRNA genes occupy an ambiguous position between host-gene annotation and retroviral transcription. For these loci, the annotated gene span largely coincides with HERV internal sequence, suggesting that at least some may represent retroviral loci captured within current lncRNA annotations rather than conventional host genes carrying incidental TE fragments. At the same time, the marked variation in exonic HERV content shows that this interpretation cannot be generalized to the mature RNA products of all loci: Some transcripts may remain predominantly HERV-derived, whereas others may combine retroviral and nonretroviral sequence.

Their limited functional annotation further underscores how little is known about this class of loci. Some may represent incidental transcription of retroviral regions, whereas others may have acquired transcript-level functions. Distinguishing these possibilities will require evidence of reproducible transcription and functional consequences, potentially complemented by analyses of evolutionary constraint.

The genome-wide identification of SPARCS-like candidates extends the antisense LTR configurations originally described in protein-coding 3′ UTRs to lncRNA terminal exons [26]. These findings highlight the widespread occurrence of antisense retroviral sequences at annotated transcript termini, identifying a broader range of genomic contexts potentially compatible with complementary RNA pairing.

The enrichment of immune-related annotations among protein-coding genes carrying SPARCS-like candidates is particularly noteworthy given the established connections between endogenous retroviral transcription, dsRNA, and innate immune activation [26,41–44]. In the original SPARCS model, the relevant LTRs occur within interferon-inducible genes, but interferon responsiveness need not depend on regulatory motifs within the LTR itself. Consistent with this distinction, we did not detect enrichment of STAT/IRF motif architectures in SPARCS-like candidate LTRs relative to other LTRs. Determining which candidates generate dsRNA and engage innate immune pathways will require strand-specific expression data, direct dsRNA detection, and locus-specific perturbation.

Several broader limitations should nevertheless be considered. The study is genome-wide and annotation-based, and candidate activity was not systematically assessed across cell types, tissues, or developmental stages. Moreover, our analyses were restricted to HERV-derived sequence; other TE classes, including LINEs (long interspersed nuclear elements) and SINEs (short interspersed nuclear elements), also contribute extensively to transcript architecture and post-transcriptional regulation [36–38], so the present analysis does not establish which of the patterns described here are specific to HERVs. In addition, “conserved” refers here to preservation of retroviral domain sequence as measured by HMM profile coverage rather than cross-species evolutionary conservation; insertion age, lineage specificity, and selective constraint were not assessed. Finally, some analyses rely on previously generated HERV domain and LTR annotations [5,6] and therefore depend on the completeness and accuracy of these underlying catalogs.

These considerations also define useful directions for follow-up. Integrating locus-resolved HERV expression with crosslinking and immunoprecipitation (CLIP)-based RBP binding data [45], ribosome profiling to investigate translational potential [46], and direct dsRNA measurements [47,48] would help establish the functional relevance of candidate HERV-derived features in specific cellular contexts. Comparative analyses across other TE classes and species could further determine which transcript architectures are HERV-specific and which represent broader properties of TE-derived sequence.

In summary, this study maps several ways in which HERV-derived sequence intersects with human transcript architecture and candidate RNA-regulatory features. HERV internal regions show a composition-controlled excess of predicted RBP motifs with subfamily-specific profiles; recognizable retroviral domain sequence is associated with unusually long terminal exons; a subset of annotated lncRNA genes largely coincides with HERV insertions; and antisense LTRs occur in widespread SPARCS-like configurations at transcript termini. Together, this genome-wide framework identifies specific loci and transcript contexts in which the RNA-level consequences of HERV-derived sequence can be tested experimentally.

## Methods

### Retrieval of HERV internal region and LTR annotations from published resources

HERV internal region sequences, provided in FASTA and BED formats together with associated internal domain annotations, were retrieved from the Zenodo repository Domain-Level Annotation and Conservation of Human Endogenous Retroviruses in the GRCh38 Genome [6,49]. HERV LTR coordinates were obtained from the Zenodo repository Structural Annotation and Transcription Factor Motif Mapping of Human Endogenous Retrovirus LTRs [5,50].

### Definition of HERV transcriptional units and locus identifiers

To facilitate consistent locus-level analyses across genomic, structural, and regulatory annotations, HERV internal regions were extended to define unified locus identifiers by incorporating proximal LTRs. Internal regions were extended to include the nearest upstream and downstream LTRs of the same subfamily and strand within 200 bp, retaining only the closest valid element on each side. Extensions that would have introduced overlaps with neighboring loci were reverted to original boundaries. The resulting loci were assigned unique identifiers and represented in gene transfer format (GTF), enabling consistent tracking of individual HERV insertions across all analyses. These locus-level identifiers were used as a unifying framework for integrating domain annotations, regulatory features, and transcript-context overlaps, whereas LTR-specific features were incorporated using external mapping resources.

### Overview of the analysis workflow

The analysis pipeline consisted of 3 main stages. First, genomic, structural, and regulatory features of HERV internal regions and LTRs were systematically annotated, particularly RBP motifs and transcript context. Second, these annotations were integrated into a unified locus-level table capturing structural, regulatory, and genomic context information for each HERV internal region. Third, transcript-level and gene-level analyses were performed to characterize the embedding of HERV sequences and conserved domains within host transcripts, including their distribution across transcript features, contribution to lncRNA architecture, and association with regulatory and functional annotations.

### Prediction of RBP motifs

RBP motifs were predicted by scanning HERV internal region sequences with position weight matrices derived from the ATtRACT database [51]. Human RBP motifs were extracted from ATtRACT and converted to MEME format [52], retaining matrix identifiers and associated gene annotations. Motifs were restricted to *Homo sapiens* and further filtered to include CisBP-RNA-derived entries [53], yielding a library of 151 motifs representing 93 RBPs; these 93 RBPs constitute the tested RBP universe of all downstream RBP analyses.

FASTA sequences of HERV internal regions were used as input for motif scanning. Background nucleotide frequencies were estimated directly from the input sequences using a first-order Markov model computed with the MEME Suite (version 5.5.9) tool fasta-get-markov under strand-specific conditions to match the transcript-oriented scanning strategy. This single background, estimated from the observed sequences, was used for all observed and shuffled scans described below.

Motif scanning was performed with FIMO [54] using a primary *P*-value threshold of *P* ≤ 10^−4^ and a maximum of 10^6^ stored matches. Scans were conducted in a strand-specific manner (--norc), treating sequences as transcript-oriented and avoiding reverse-complement scoring. FIMO output was parsed to recover genomic coordinates of motif occurrences from sequence identifiers encoding chromosomal position and strand. Coordinates were converted from sequence-relative positions (1-based inclusive) to genomic BED coordinates (0-based, end-exclusive), accounting for sequence orientation. The resulting dataset comprised genome-wide maps of predicted RBP motifs across HERV internal regions, including motif identity, genomic coordinates, strand, and statistical significance metrics.

### Composition-controlled RBP motif enrichment

To estimate the expected motif burden, we generated 5 dinucleotide-preserving shuffled sequences per HERV internal region using a Eulerian-path-based algorithm. Shuffling preserved sequence length, mono- and dinucleotide counts, and terminal bases, treating ambiguous nucleotides (N) as ordinary symbols. These properties were verified for all loci and replicates. Observed and shuffled sequences were scanned with FIMO using identical parameters, motif libraries, and first-order background models.

Motif occurrences were counted per HERV locus and RBP, including zero counts for loci without hits. Expected counts were calculated as the mean across 5 shuffled replicates. Motif density was expressed as occurrences per kilobase, and observed/expected ratios were calculated relative to the mean shuffled motif burden. Global and subfamily densities were calculated from total motif counts and sequence lengths, while shuffled median locus densities were averaged across replicates. Observed and expected locus-level densities were compared across all 53,176 HERV internal regions using a paired 2-sided Wilcoxon signed-rank test.

For each of the 104 HERV subfamilies and 93 RBPs, observed per-locus motif counts were compared with the mean shuffled counts using a paired 2-sided Wilcoxon signed-rank test. Combinations with fewer than 5 loci or no nonzero paired differences were excluded. *P* values were adjusted across all subfamily × RBP tests using the BH procedure, with significance defined as globally adjusted *P* < 0.05. Significant RBPs were classified as enriched or depleted according to the effect size log₂[(observed + 0.5)/(expected + 0.5)], calculated from subfamily-level totals; the pseudocount was used only for this calculation. Within-subfamily BH-adjusted *P* values were also calculated but not used for classification (Table S1).

### Functional analysis of RBP motif profiles

GO BP overrepresentation analysis was performed separately for RBPs significantly enriched in HERVH and HERVK using clusterProfiler (enrichGO; clusterProfiler [55] v4.10.1, org.Hs.eg.db and GO.db v3.18.0), with all 93 tested RBPs as the background universe and gene-set sizes of 10 to 500. To assess functional differences across the complete motif profiles, all 93 RBPs were ranked by the difference in their log₂ observed/expected ratios (HERVH minus HERVK) and analyzed using pre-ranked gene set enrichment analysis (GSEA) with fgsea [56] (v1.28.0; fgseaMultilevel), considering GO BP gene sets containing 5 to 50 tested RBPs. *P* values were adjusted using the BH procedure within each analysis, with adjusted *P* < 0.05 considered significant. Negative normalized enrichment scores indicated HERVK-biased enrichment (Table S2).

### Sensitivity analysis at a stricter FIMO threshold

To assess sensitivity to motif-calling stringency, observed and shuffled FIMO outputs were filtered to retain motif calls with *P* ≤ 10^−5^, without rescanning. The analyses were repeated using the same methods as the primary analysis, including motif burden, subfamily × RBP enrichment with global BH correction across the same test set, GO overrepresentation analysis, and pre-ranked GSEA. The original threshold (*P* ≤ 10^−4^) remained the primary analysis, with the stricter threshold used only for sensitivity assessment (Table S3).

### FMR1 motif-guided locus screen

To prioritize HERVH loci carrying predicted FMR1 motifs, motif calls from the primary FIMO analysis were pooled across FMR1 matrices and filtered at *P* ≤ 10^−5^. Loci containing at least 2 motifs were retained and ranked by motif density (occurrences/kb). HERVH-int_00795 was manually selected for integrative inspection based on embryonic RNA-seq and epigenomic evidence available through the HERVarium browser.

### Annotation of HERV genomic and transcript context

The genomic and transcript context of HERV internal regions, LTRs, and associated protein domains was determined through systematic overlap with GENCODE v48 annotations [57]. Gene, transcript, exon, coding sequence (CDS), and UTR features were extracted from the GENCODE GTF file and converted to BED format.

Genomic overlaps between HERV internal regions, retroviral protein domains, LTR elements, and transcript features were computed with bedtools [58] (v2.31.1) on BED files sorted according to GRCh38 chromosome order.

Gene-level exons were defined by merging overlapping or adjacent exons across all transcripts of each gene. Introns were defined as the portions of each gene body not covered by its exons. Overlaps between HERV internal regions and genomic features (genes, exons, introns, CDS, and UTRs) were computed using bedtools intersect in both strand-independent and strand-specific modes. Strand-specific intersections were used to identify HERV insertions co-oriented with host transcripts, which is particularly relevant for transcript incorporation analyses.

For retroviral protein domains, overlaps with transcript-resolved exon, CDS, and UTR annotations were computed using strand-aware base-pair-resolution intersections (bedtools intersect -wo). Overlapping intervals were merged prior to quantification to avoid double counting and to derive unique overlap lengths. Domain–feature overlap fractions were then calculated relative to total domain length.

UTRs were subdivided into 5′ and 3′ components in a transcript-aware manner using CDS boundaries and transcript strand, with CDS start and end coordinates used to assign UTR segments according to transcript orientation. Terminal exons were defined from transcript-resolved exon coordinates using strand-aware exon ordering.

For LTR elements, transcript-feature overlaps were computed in a strand-aware manner to identify antisense insertions relative to host transcripts, enabling detection of genomic configurations compatible with dsRNA formation. Proximity relationships between HERV internal regions and annotated genes or transcription start sites were computed using bedtools closest, both with and without strand constraints.

### Construction of an integrated annotation table for HERV internal regions

To enable downstream analyses, a comprehensive integrated annotation table was constructed for all HERV internal regions by combining genomic, structural, regulatory, and transcript-context features into a unified framework.

For each internal region, retroviral protein domain annotations were aggregated at the locus level, retaining domain identity, domain class (e.g., GAG, POL, and ENV), and conservation metrics. LTR-associated information was incorporated by linking each internal region to its corresponding 5′ and 3′ LTRs using the file HERV_internal_LTR_map.tsv from the Zenodo repository Structural Annotation and Transcription Factor Motif Mapping of Human Endogenous Retrovirus LTRs. Integrated LTR features included LTR length, transcription factor binding motif burden, and reconstruction confidence.

RBP motif information was summarized per locus by aggregating motif occurrences identified by FIMO, including both total motif burden and the number of unique RBPs associated with each internal region. Genomic context annotations were integrated from overlap and proximity analyses, including compact summaries of overlapping genomic features and associated gene biotypes. The final integrated table therefore captured, for each HERV internal region, its structural features, regulatory potential, and genomic context, and included the locus-level identifiers defined above to enable consistent tracking across analyses. This table served as the main input for downstream transcript- and gene-level analyses.

### Conserved HERV domains in transcript context and terminal-exon analyses

HERV protein-domain predictions were intersected with same-strand GENCODE transcript exons. Domain–transcript associations were retained when at least 80% of the domain overlapped exonic sequence, including all transcript isoforms meeting this criterion. Domains with HMM profile coverage ≥0.40 were classified as conserved. A domain was considered terminal in a transcript when at least 80% of its length overlapped the terminal exon. Terminal localization was assessed across these transcripts and separately for multi-exon transcripts.

To determine whether terminal localization exceeded that expected from transcript architecture, we constructed a transcript-aware positional null model. For each conserved domain, all possible genomic placements of an interval of the same length were evaluated while keeping its chromosome, strand, and associated transcripts fixed. Valid placements required at least 80% exonic overlap, and terminal localization was defined using the same ≥80% terminal-exon overlap criterion applied to observed domains. Assuming independence among domains, domain-specific probabilities were combined using an exact Poisson-binomial distribution to calculate expected counts, 95% null intervals, and one-sided *P* values. The analysis was repeated for multi-exon transcripts. Robustness was assessed using alternative terminal-localization criteria (any terminal-exon overlap or terminal localization in all qualifying transcripts) and by extending the analysis to all isoforms of the associated genes. Monte Carlo simulations (10,000 iterations) were used to verify the exact null calculations.

To assess the relationship between domain preservation and terminal localization, conserved (HMM profile coverage ≥0.40) and lower-coverage domains were compared using 2-sided Fisher’s exact tests. To account for multiple domains originating from the same HERV insertion, the analysis was repeated using only the highest-coverage domain per HERV locus.

Terminal-exon architecture was analyzed in multi-exon transcripts, excluding single-exon transcripts. Transcripts were classified according to whether they contained conserved HERV domains in terminal exons (≥80% overlap), contained conserved domains only in nonterminal exons, or belonged to background genes without qualifying conserved domains. Other isoforms of domain-containing genes were classified separately. Terminal-exon lengths were compared using 2-sided Mann–Whitney U tests, with Cliff’s delta as effect size and BH correction for multiple testing. Comparisons were performed overall, separately for lncRNAs and protein-coding genes, and at the gene level using median and maximum terminal-exon lengths.

### Quantification of HERV contribution to lncRNA genes

The contribution of HERV internal regions to lncRNA architecture was quantified by integrating genomic overlap information with transcript annotation data. HERV internal regions overlapping lncRNA genes on the same strand were first identified from genome-wide intersection analyses. For each lncRNA gene, all overlapping HERV loci were collected.

To enable accurate base-pair–level quantification of HERV contribution, gene body and exon intervals were reconstructed directly from GENCODE v48 annotations. Gene body regions were defined using full gene span coordinates, and exon intervals were merged per gene to generate nonredundant exon representations.

HERV contribution to lncRNA genes was quantified by calculating the proportion of each gene body and its merged exonic sequence overlapping same-strand HERV internal regions, avoiding double-counting overlapping intervals. Based on gene-body coverage, lncRNAs were classified into 4 categories: ≤10%, >10% to 50%, >50% to 80%, and >80% HERV coverage. Gene-body coverage includes both exonic and intronic sequence, whereas exonic coverage was calculated separately. The same coverage measures were applied to protein-coding genes (Tables S4 and S5).

### Functional annotation and literature mining of HERV-associated lncRNAs

To characterize the functional landscape of HERV-associated lncRNAs, annotations from curated databases and large-scale literature mining were integrated.

Functional annotations were retrieved from LncRNAWiki [29]. Genes were matched by Ensembl gene identifiers, gene symbols, and known synonyms, with normalization steps applied to harmonize naming conventions. Matched annotations were aggregated at the gene level to summarize reported functional mechanisms, biological processes, molecular functions, pathway associations, and disease links. In parallel, GO annotations [30,31] were retrieved from Ensembl BioMart [59,60] and summarized per gene.

To complement curated resources, functional evidence was extracted directly from the biomedical literature through large-scale text mining. PubMed abstracts were retrieved via the National Center for Biotechnology Information (NCBI) Entrez API [61] using lncRNA-related queries. Abstracts published from 2000 onward were downloaded together with associated metadata, including PMID, year, and title.

Natural language processing was performed using spaCy [62]. Candidate lncRNA mentions were detected using regular expression patterns capturing common naming conventions, including LINC identifiers, antisense transcript names, and Ensembl gene IDs. Candidate names were normalized and filtered to remove ambiguous or noninformative terms.

For each sentence containing candidate lncRNA mentions, functional context was inferred through predefined keyword sets capturing mechanistic, phenotypic, associative, pathway-related, and regulatory terms. Sentences were classified into categories such as mechanistic evidence, perturbation-based functional evidence, associative observations, or list-like descriptions. A tiered evidence score was assigned, prioritizing mechanistic and perturbation-based statements over descriptive or associative evidence.

Only informative sentences containing both candidate lncRNA mentions and relevant functional terms were retained. For each lncRNA–function association, structured information including functional keywords, sentence class, evidence tier, and the original sentence was extracted. Results were aggregated across abstracts to generate a gene-level dataset of literature-derived functional evidence.

Finally, LncRNAWiki, GO, and literature-derived annotations were integrated into a unified framework, enabling classification of HERV-associated lncRNAs according to the presence and type of functional support (Table S6).

### Identification of SPARCS-like antisense LTR configurations

SPARCS-like configurations were identified by integrating HERV LTR annotations with transcript-context information derived from GENCODE. Candidate loci were defined as LTR insertions occurring in antisense orientation relative to host transcripts and located within transcript termini, specifically within 3′ UTRs of protein-coding genes or within terminal exons of lncRNAs [26]. To avoid redundancy arising from multiple transcript isoforms, candidates were collapsed to unique LTR instances based on genomic coordinates while preserving aggregated information on associated genes and transcripts.

The resulting SPARCS-like set was further integrated with LTR structural annotations derived from U3–R–U5 segmentation analyses retrieved from the Zenodo repository Structural Annotation and Transcription Factor Motif Mapping of Human Endogenous Retrovirus LTRs. For each LTR, incorporated structural features included the presence of reconstructed U3 and U5 regions, and overall annotation confidence. This enabled comparison of SPARCS-like LTRs against all other annotated LTRs.

Differences in structural integrity and annotation confidence between SPARCS-like and background LTRs were evaluated statistically. Fisher’s exact tests were used for categorical comparisons, including proportions of successfully reconstructed elements, and Wilcoxon rank-sum tests were used for continuous measures such as confidence scores.

### Analysis of interferon-responsive regulatory features in LTRs

To characterize interferon-related regulatory potential in HERV LTRs, transcription factor binding motifs were analyzed using previously computed FIMO scans across all annotated LTR sequences, retrieved from the Zenodo repository Structural Annotation and Transcription Factor Motif Mapping of Human Endogenous Retrovirus LTRs. Analyses focused on STAT1::STAT2 heterodimer motifs, representing type I/III interferon signaling, STAT1 motifs, representing type II interferon signaling, and IRF family motifs [33–35].

To obtain robust motif counts, overlapping and redundant hits within each LTR were collapsed using a minimum overlap threshold of 50%, ensuring that each predicted binding site was represented only once.

Local motif clustering was assessed using 75-bp windows anchored at motif-site midpoints, jointly considering STAT and IRF motifs to identify colocalized regions. For each LTR, the densest window of combined STAT and IRF motifs was recorded together with the number of STAT and IRF sites it contained.

LTRs were then assigned to categories reflecting their predicted interferon-responsive potential based on the composition of the densest window. LTRs containing at least one STAT motif and one IRF motif within the same window were classified as having synergistic potential and further subdivided into very high (≥3 total motifs within the window) and high (exactly one STAT and one IRF motif within the window) categories. LTRs exhibiting strong clustering of a single motif class (≥3 STAT or ≥3 IRF motifs within a window) were also classified as high. LTRs with exactly 2 motif occurrences of the same class within the window were classified as medium, whereas those with a single motif occurrence were classified as low. This classification provided a proxy for the presence of cooperative regulatory modules consistent with interferon-responsive enhancers [33,35,63].

SPARCS-like LTRs were intersected with interferon motif annotations to assess whether dsRNA-compatible genomic configurations are associated with canonical interferon-responsive regulatory architectures. Enrichment of interferon-associated motif classes was evaluated at the LTR level using Fisher’s exact tests, comparing the proportion of LTRs classified as interferon-responsive between SPARCS-like candidates in protein-coding 3′ UTRs and all other motif-scanned LTRs, with BH correction. Additional analyses focused on synergistic architectures corresponding to colocalized STAT and IRF motifs.

### GO, MSigDB, and transcription factor motif enrichment of SPARCS-like candidates

GO BP overrepresentation analysis was performed using clusterProfiler (enrichGO) for protein-coding genes containing SPARCS-like LTRs in their 3′ UTRs, using GENCODE v48 protein-coding genes as the background universe. Gene sets containing 10 to 500 genes were considered. Immunologic signature enrichment was assessed using clusterProfiler (enricher) against the MSigDB [32] C7 collection (release 2026.1.Hs), with the corresponding mapped protein-coding gene universe as background. Both analyses applied BH correction, retaining terms with adjusted *P* ≤ 0.05 and *q* ≤ 0.20 (Table S7).

Transcription factor motif enrichment was evaluated separately for SPARCS-like LTRs in protein-coding 3′ UTRs and lncRNA terminal exons, using 730 JASPAR [64] CORE motifs. Motif presence was compared between candidate LTRs and all other motif-scanned LTRs using 2-sided Fisher’s exact tests. LTRs without motif hits were considered motif-negative. Motifs were classified as enriched or depleted according to their odds ratios when the BH-adjusted *P* was below 0.05.

GO BP overrepresentation analysis was additionally performed for transcription factors associated with enriched or depleted motifs, using the tested motif library as background. These results were considered exploratory, with terms reported at adjusted *P* ≤ 0.10 and *q* ≤ 0.20 (Table S8).

## Data Availability

The human reference genome assembly (GRCh38) was obtained from the GENCODE project (release 48; https://ftp.ebi.ac.uk/pub/databases/gencode/Gencode_human/release_48/GRCh38.primary_assembly.genome.fa.gz). HERV internal region annotations were retrieved from the Zenodo repository Domain-Level Annotation and Conservation of Human Endogenous Retroviruses in the GRCh38 Genome (https://doi.org/10.5281/zenodo.17662456), including the files HERV_internal_v5.bed, HERV_internal_sequences_v5.fasta, HERV_domains_v5.bed, and HERV_loci_annotated_domains_v5.tsv. HERV LTR annotations were obtained from the Zenodo repository Structural Annotation and Transcription Factor Motif Mapping of Human Endogenous Retrovirus LTRs (https://doi.org/10.5281/zenodo.18554805), including the files HERV_ltr.bed, HERV_internal_LTR_map.tsv, HERV_LTR_U3_R_U5_catalogue.tsv, and HERV_LTR_fimo_results_tsv.tar.xz. RBP motifs were obtained from the ATtRACT database (https://attract.cnic.es), available at https://attract.cnic.es/attract/static/ATtRACT.zip. All HERV annotations generated in this study, together with their description, are publicly available in Zenodo (https://doi.org/10.5281/zenodo.23361077). All code used for data processing and analysis in this study is available as a collection of Python, Bash, and R scripts at https://github.com/funcgen/herv-rna-regulation.
