# Supplementary material for: Genome-Wide Characterization of HERV-Derived RNA Regulatory Potential in the Human Transcriptome
Source: Comput Struct Biotechnol J. 2026 Nov 5;35(1):0244. doi: 10.34133/csbj.0244 (PMC13735202; doi:10.34133/csbj.0244)
Supplement: Supplementary file 1 [file csbj.0244.f1.zip › supp_figures.docx]

A

B

**Supplemental Figure 1. Conserved HERV-derived domains in lncRNA loci and regulatory context.** (A–B) Genome-browser views of representative lncRNA loci harboring conserved HERV-derived domains within terminal exons. Tracks show HERV internal regions, annotated retroviral protein domains, GENCODE gene models, and ENCODE candidate cCREs. In both examples, conserved domains (including protease, RNase H, reverse transcriptase, and Env) are embedded within transcriptionally active regions and coincide with multiple cCREs, indicating integration of retroviral coding remnants within regulatory genomic environments.

**Supplemental Figure 2. Regulatory landscape of SPARCS-like LTRs.** Gene Ontology (GO) enrichment analysis of transcription factors depleted in SPARCS-like LTRs located within lncRNA transcripts. Enriched terms are predominantly associated with developmental processes. Dot size represents gene count and color indicates adjusted p-value.
