# Supplementary material for: Genome-Wide Characterization of HERV-Derived RNA Regulatory Potential in the Human Transcriptome
Source: Comput Struct Biotechnol J. 2026 Nov 5;35(1):0244. doi: 10.34133/csbj.0244 (PMC13735202; doi:10.34133/csbj.0244)
Supplement: Supplementary file 1 [file csbj.0244.f1.zip › supp_tables_legend.docx]

**Supplemental Table 1. Composition-controlled enrichment of predicted RBP motifs across HERV subfamilies.** Observed motif occurrences were compared with five dinucleotide-preserving shuffled controls at the primary FIMO threshold (P ≤ 10-4). The table provides observed and expected motif burdens, log₂ observed/expected ratios, and globally Benjamini–Hochberg-adjusted P values for HERVH, HERVK, and all 104 tested HERV subfamilies, including a summary of significant RBP associations.

**Supplemental Table 2. GO Biological Process enrichment of HERVH- and HERVK-biased RBP motif profiles.** Pre-ranked gene-set enrichment analysis of 93 RBPs ranked by the difference in composition-controlled log₂ observed/expected ratios between HERVH and HERVK, together with the underlying RBP ranking. Results include enrichment scores, normalized enrichment scores, adjusted P values, and leading-edge RBPs. Negative enrichment scores indicate HERVK-biased associations.

**Supplemental Table 3. Sensitivity of RBP motif enrichment to FIMO threshold stringency.** Results obtained with the primary (P ≤ 10-4) and stricter (P ≤ 10-5) motif-calling thresholds, including global and subfamily-specific motif burdens, changes in RBP enrichment and depletion, and GO over-representation and pre-ranked enrichment analyses.

**Supplemental Table 4. HERV internal-region contribution to annotated lncRNA genes.** Gene-body and exonic HERV sequence coverage for 4,074 lncRNA genes overlapping same-strand HERV internal regions. Reported information includes gene identifiers, genomic coordinates, overlap statistics, and associated HERV subfamilies.

**Supplemental Table 5. HERV internal-region contribution to annotated protein-coding genes.** Gene-body and exonic HERV sequence coverage for 1,521 protein-coding genes overlapping same-strand HERV internal regions. Reported information includes gene identifiers, genomic coordinates, overlap statistics, and associated HERV subfamilies.

**Supplemental Table 6. Functional annotation of HERV-associated lncRNA genes.** Gene-level functional annotations for 4,074 lncRNAs overlapping HERV internal regions, integrating LncRNAWiki, Gene Ontology and literature-derived evidence. The table includes reported functional categories, supporting PubMed identifiers, and gene-body HERV coverage.

**Supplemental Table 7. SPARCS-like antisense LTR candidate catalogue and associated analyses.** The table provides genomic and transcript annotations for antisense LTR insertions within protein-coding 3′ UTRs and lncRNA terminal exons, together with GO Biological Process and MSigDB C7 enrichment results for associated protein-coding genes, interferon-responsive motif analyses, and comparisons of LTR structural integrity. Individual LTRs may have multiple transcript associations.

**Supplemental Table 8. Transcription factor motif enrichment in SPARCS-like LTRs associated with lncRNAs.** Enrichment and depletion of predicted transcription factor motifs in antisense LTRs overlapping lncRNA terminal exons relative to other motif-scanned LTRs. The table reports Fisher’s exact test results, multiple-testing-adjusted P values, and exploratory GO Biological Process enrichment of transcription factors associated with depleted motifs (adjusted P ≤ 0.10).
